# Deciphering the Contribution of BP230 Autoantibodies in Bullous Pemphigoid

**DOI:** 10.3390/antib11030044

**Published:** 2022-06-28

**Authors:** Connor Cole, Luca Borradori, Kyle T. Amber

**Affiliations:** 1 Division of Dermatology, Rush University Medical Center, Chicago, IL 60612, USA; kyle_amber@rush.edu; 2 Department of Dermatology, Inselspital, Bern University Hospital, University of Bern, 3010 Bern, Switzerland; luca.borradori@insel.ch; 3 Department of Internal Medicine, Rush University Medical Center, Chicago, IL 60612, USA

**Keywords:** bullous pemphigoid, BP230, autoimmune blistering disease, immunobullous disease, dystonin

## Abstract

Bullous pemphigoid (BP) is a subepidermal autoimmune blistering disease predominantly affecting elderly patients and carries significant morbidity and mortality. Patients typically suffer from severe itch with eczematous lesions, urticarial plaques, and/or tense blisters. BP is characterized by the presence of circulating autoantibodies against two components of the hemidesmosome, BP180 and BP230. The transmembrane BP180, also known as type XVII collagen or BPAG2, represents the primary pathogenic autoantigen in BP, whereas the intracellular BP230 autoantigen is thought to play a minor role in disease pathogenesis. Although experimental data exist suggesting that anti-BP230 antibodies are secondarily formed following initial tissue damage mediated by antibodies targeting extracellular antigenic regions of BP180, there is emerging evidence that anti-BP230 IgG autoantibodies alone directly contribute to tissue damage. It has been further claimed that a subset of patients has a milder variant of BP driven solely by anti-BP230 autoantibodies. Furthermore, the presence of anti-BP230 autoantibodies might correlate with distinct clinical features. This review summarizes the current understanding of the role of BP230 and anti-BP230 antibodies in BP pathogenesis.

## 1. Introduction

Bullous pemphigoid (BP) is the most common autoimmune subepidermal blistering disease and is generally seen in elderly patients. The typical clinical manifestations of BP include eczematous lesions and urticarial plaques in variable combination with tense blisters which predominantly develop on the trunk and the proximal region of the upper and lower limbs. Atypical forms without obvious blistering are observed in up to 20% of cases [1,2]. Patients also typically experience severe pruritus. Mucosal involvement, almost invariably limited to the oral cavity, can be seen in nearly one-fifth of patients [2,3,4].

Immunologically, the disease is characterized by the presence of circulating and tissue-bound IgG autoantibodies directed against BP180 and BP230. Autoantibodies of the IgE and IgA class are also less frequently detectable. The two target antigens, BP180 (a transmembrane collagenous protein, also called type XVII collagen or BPAG2) and BP230 (a cytoplasmic protein of the plakin family) are important components of hemidesmosomes, which promote dermo-epidermal adhesion and epidermal integrity.

The diagnosis of BP relies on clinical and immunopathological findings. While light microscopy studies of blistered skin specimens characteristically demonstrate subepidermal blister formation with an infiltrate rich in eosinophils, histopathological findings may be often nonspecific and are invariably not sufficient to diagnose BP [1]. Direct immunofluorescence microscopy studies, which typically demonstrate linear deposits of IgG and/or C3 along the basement membrane zone, as well as immunoserological tests, are usually required and necessary for its diagnosis. ELISAs to search for circulating autoantibodies against BP180 and/or BP230 or indirect immunofluorescence studies using NaCl-separated normal human skin are very useful for the proper classification and diagnosis of patients with suspected BP. Systemic oral corticosteroids or high potency topical corticosteroids are regarded as the first-line of therapy for both moderate and severe disease. The disease has significant mortality and well-documented associations with several neurological conditions [5].

There are ample data indicating that BP180 plays a primary role in the pathogenicity of BP, with nearly 90% of BP patients having IgG autoantibodies targeting the extracellular membrane proximal region of BP180, termed the NC16A domain. However, as many as 80% and 68% of BP patients also have circulating anti-BP230 IgG and IgE autoantibodies, respectively [6,7,8,9,10,11,12,13,14,15,16,17].

Although the exact pathogenic role of the cytoplasmic protein BP230 in BP pathogenesis has not yet been fully elucidated, results obtained from several mouse models of BP strongly suggest that autoantibodies against BP230 alone directly contribute to tissue damage and blister formation [18,19,20,21,22]. In addition, it is likely that the serological profile affects the clinical features observed in BP [23]. This review will summarize current knowledge about BP230 and anti-BP230 antibodies and their relevance in BP pathogenesis.

## 2. BP230 Structure and Expression

BP230, also known as bullous pemphigoid antigen 1 is a 230 kDa intracellular protein [24]. It was the first protein that was identified as a target by circulating autoantibodies from BP sera as assessed by immunoprecipitation studies. BP230/BPAG1e represents the epithelial protein isoform encoded by the dystonin (*DST*) gene (hence referred to as BPAG1e). The other major isoforms BPAG1a and BPAG1b are predominantly expressed in the brain and skeletal muscle, respectively.

**Figure 1:**
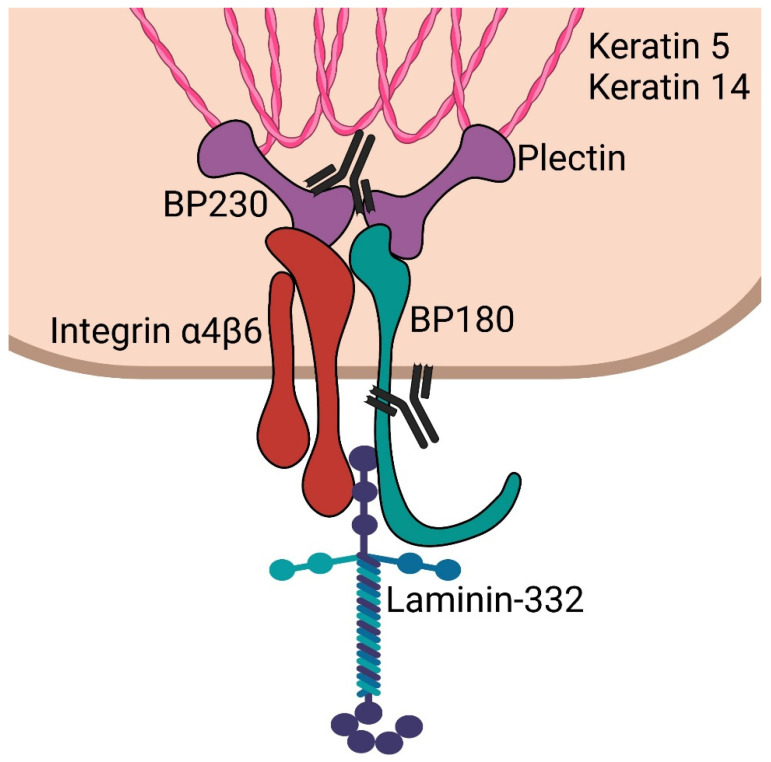
Schematic of epidermal hemidesmosome. BP230 and plectin bind to keratin intermediate filaments. These subsequently complex with integrin α4β6 and BP180 (collagen 17). These then interact with laminin-332 in the lamina lucida. Antibodies are shown targeting the C-terminus of BP230 and the NC16a domain of BP180, which are most often targeted in bullous pemphigoid.

BP230/BPAG1e is an important component of hemidesmosomes, junctional adhesion complexes present in the epidermis and other stratified squamous epithelia, including the cornea. BP230/BPAG1e is a member of the plakin family of cytolinkers, such as desmoplakin and plectin [25,26,27]. It contains an N-terminus followed by a spectrin repeat, a plakin domain, a coiled-coil rod domain, two plakin repeat domains, and a C-terminal extremity [28]. The N-terminal portion of the protein interacts with the α6β4 integrin and with BP180 (Figure 1). The C-terminal regions encompassing the plakin repeat domain and the C-extremity mediate the binding of BPAG1e to the epidermal specific intermediate filament K5/K14 and K6/K17. These interactions are critical in promoting stable adhesion between the epidermal cells and the basement membrane as well as cytoskeletal architecture [29,30]. *DST*-knockout mice develop discrete signs of skin blistering as a result of basal keratinocyte fragility with cytoskeletal disruption [31]. In addition, these mice demonstrate degeneration of sensory nerves with severe dystonia as well as skeletal muscle defects. These observations confirm the important role of the various BPAG1 isoforms in the maintenance of keratinocyte, skeletal muscle, and neuronal cell homeostasis and resilience.

BPAG1a and BPAG1b constitute the two other major isoforms encoded by the DST gene, showing a similar domain organization. BPAG1a has a predicted molecular mass of 625 kDa. In mice, it has been found to be predominantly expressed in the brain, as well as the lung, liver, kidney, ovary, spleen, and testis. BPAG1b is 834 kDa and shares a very similar structure with BPAG1a with the exception of one region between the plakin domain and spectrin repeats which contains two plakin repeat domains and two spectrin repeats. This isoform is expressed in the myocardium and skeletal muscle of mice, as well as the liver, ovary, testis, spleen, vertebral cartilage, and tongue epithelium [32,33]. It is important to note that no data for specific isoform expression in humans have been obtained as of yet. The functions of BPAG1a/b on the cellular level include interactions with microtubules, vesicular transport, effects on Golgi positioning, and regulation of the actin network [34,35]. Pathogenic variants in *DST* which selectively affect the BPAG1a/b isoforms in humans can result in severe neurological diseases including encephalopathy, delayed visual maturation, and mental retardation [36].

## 3. The Pathogenic Contribution of BP230 Autoantibodies

There are compelling data that support the role of BP180 in the pathogenesis of BP. Several in vitro and in vivo studies have shown ample evidence that the binding of anti-BP180 autoantibodies to their target antigen results in the activation of the complement cascade and granulocytes [37,38,39,40,41]. Although circulating autoantibodies recognize several antigenic regions throughout the BP180 molecule, the NC16A domain, located in the extracellular cell membrane proximal portion of BP180, appears to be the immunodominant region. The latter is recognized by 85% of patients’ sera [7,42,43,44].

In contrast to the transmembrane BP180, the intracellular BP230 antigen was originally thought to play a minor pathogenic role. However, anti-BP230 antibodies are found in the majority of BP patients [13,35]. Although several studies have investigated their contribution to the BP disease process, the pathogenic relevance of anti-BP230 antibodies has been a matter of debate for decades [18,19,45]. In 2003, Kiss et al. found that passive transfer of anti-BP230 IgG can induce clinical and immunopathological features of BP in neonatal mice [19]. Another study demonstrated that rabbits immunized with BP230-derived peptides developed antibodies that could deposit on the BMZ and trigger an enhanced inflammatory response following UVB-induced epithelial injury [18]. Two recent studies by Haeberle et al. and Muramatsu et al. provide additional evidence supporting the pathogenicity of BP230. They demonstrated the production of anti-BP230 IgG autoantibodies in mice with dysfunctional regulatory T cells [20,21]. Haeberle et al. also reported that the monoclonal antibody 20B12 against BP230 is capable of inducing subepidermal blistering in neonatal mice [20]. However, this model lacked eosinophilic infiltrate in the BMZ and thus did not replicate the characteristic histology of BP. This is, however, generally the case in passive transfer murine models of BP.

A novel tissue-specific conditional knockout mouse model has been very useful to gain significant insight into the effect of autoimmunity against BP230 [46]. To investigate the role of BPAG1 antibodies in the pathogenicity of BP, Makita et al. generated a mouse model with the conditional knockout of BPAG1 confined to keratin-5 expressing stratified epithelial cells. These mice were then immunized against the C-terminal portion of BP230. The latter is thought to contain the immunodominant antigenic regions that are recognized by anti-BP230 antibodies in BP. Splenocytes from these mice were then transferred into immunodeficient mice. These recipient mice developed a BP-like phenotype displaying scaling and erosions on multiple areas of skin as well as histologically, subepidermal blistering. In addition, direct immunofluorescence studies showed IgG deposition along the dermo-epidermal junction. The study additionally found that skin wounding resulted in increased blistering in these mice. Overall, these findings provided strong evidence that antibodies to BP230, and specifically autoreactivity of the C-terminal domain, may have a pathogenic role in the development of tissue damage and blister formation in BP.

On the other hand, Feldrihan et al. reported that rabbit polyclonal anti-BP230 IgG antibodies subcutaneously transferred into neonatal and adult mice did not induce experimental BP [45]. In this study, however, the transferred anti-BP230 antibodies were not able to activate the complement system in vivo, an observation most likely explaining the lack of obvious clinical effect. The latter study has additional important limitations. For example, since the employed mice expressed their own native BP230 it is conceivable that they developed immune tolerance to the injected antibodies. Furthermore, the autoantibodies may require a separate trigger or tissue damage resulting in the exposure of the intracellular BP230 antigen to either cause or aggravate disease in the used mouse strains. Finally, longer administration and observation times than those used by the authors may be needed to reach the disease threshold. Feldrihan et al. suggested that BP230 could support ongoing disease initiated by the granulocyte and complement activation of BP180 antibodies, or that BP230 could trigger disease via non-inflammatory mechanisms.

As previously noted, IgE autoantibodies against BP230 are also present in a significant number of BP patients. While anti-BP180 IgE antibodies have more robust mechanistic data [47,48], the presence of anti-BP230 IgE autoantibodies has been associated with increased local eosinophil recruitment. Additionally, the presence of BP230 IgE autoantibodies has been associated with increased resistance to topical corticosteroids [49]. Given its intracellular localization, it remains unclear whether anti-BP230 IgE autoantibodies drive similar immune activation and granulocyte mediated blistering as anti-BP180 IgE.

## 4. Synergistic Effect of BP230 Autoantibodies with BP180 Autoantibodies in Bullous Pemphigoid

Several mechanisms for the generation of antibodies against BP230 and their role in BP have been described. It has been proposed that anti-BP230 autoantibodies develop in the course of the disease as a result of an intermolecular epitope spreading phenomenon. This refers to an immune response that develops to a secondary epitope(s) that is distinct and not cross-reactive with the epitope which initially caused the disease [50]. In the case of BP, antibodies targeting extracellular epitopes on BP180 may induce cellular damage which leads to the exposure of the intracellular BP230 as well as to the intracellular BP180 cytoplasmic domain [8,51,52]. Autoantibodies to BP230 could then form throughout the clinical course of the disease. Multicenter studies have shown that epitope spreading events in BP primarily occur during the early stage of the disease and are associated with increased disease severity [51,53]. The latter study also suggested that antibody response to BP230 occurred after BP180 reactivity in a subset of patients [53]. Another mouse model study from 2010 focusing on the dynamics of the IgG response to BP180 found that the development of antibodies targeting extracellular epitopes preceded IgG binding to intracellular epitopes [54]. This evidence further supports the concept of a primary pathogenic extracellular epitope (BP180 ectodomain) causing tissue damage and subsequent IgG recognition of BP230 [29]. There are reports suggesting that the N-terminal region of BP230 may be the initially recognized region following basal membrane damage, followed by the C-terminal region which then becomes another immunodominant antigenic portion [8,9,10,55].

## 5. BP230 Autoantibodies as Only Mediators and Triggers of Bullous Pemphigoid?

There is also evidence that a subset of BP cases may be primarily driven by an immune response to BP230. Hayakawa et al. performed a study dividing BP patients into three groups depending on if their sera displayed reactivity with BP180, BP230, or both [23]. They identified 41 out of 153 patients whose sera reacted with only BP230. These so-called BP230-BP patients displayed lower clinical severity and better response to treatment when compared to the BP180-BP and BP180-BP230-BP groups. Based on this data, they proposed to designate this group of BP patients as having anti-BP230-type BP, corresponding most likely to a milder form of BP. The study by Hayakawa et al. is nevertheless not only limited by its retrospective nature, but also by the lack of a comprehensive in-depth characterization of the immunological profile of affected patients by different complementary technical approaches. For example, patients’ reactivity with BP180 was only studied using a commercially available ELISA-BP180 which only contains the NC16A domain. The other available data are less robust. One case report described a patient who displayed only anti-BP230 IgG and IgE during early disease and later developed anti-BP180 IgG as the disease progressed [6]. Inoue et al. also identified a patient with mucous membrane pemphigoid who had isolated anti-BP230 IgG present with no reactivity to BP180 throughout their disease course [56]. Additionally, psoriasis patients appear to be at increased risk for the development of BP by exposing BP230 [57]. However, there are several other possible mechanisms that could explain the increased risk of developing pemphigoid diseases among psoriasis patients, including local inflammation, increased activation of Th17 cells, and the production of neutrophil chemoattractants and subsequent metalloprotease release, laminin degradation, and accelerated epidermal senescence [58].

In brief, while most BP cases may be caused by initial reactivity to BP180 and progression via tissue damage and epitope spreading, some cases may develop by means of different mechanisms in which BP230 is the primary pathogenic target. It is conceivable that damage to the basal cell layer leads to the extracellular exposure of BP230 with a first immune response to BP230 which is then followed by the generation of antibodies binding to extracellular epitopes of BP180 and the activation of inflammatory cells and the complement cascade. This idea is indirectly supported by Hall et al. who provided evidence that anti-BP230 antibodies are capable of inducing subepidermal blister formation in injected experimental rabbits only upon initial tissue damage and inflammation induced by UVB light [14].

## 6. Associations of BP230 Autoantibodies with Clinical Characteristics

Although experimental evidence now exists indicating that autoantibodies to BP230 contribute to tissue damage in animal models, it is as of yet unclear whether the presence of such autoantibodies affects and has an impact on the clinical features and course in patients. For example, it has been claimed that patients with anti-BP230 antibodies have distinct clinical characteristics, such as mucosal involvement, nodular and prurigo-like lesions, neuropsychiatric comorbidity, or even increased mortality [52]. In apparent contrast, other studies have shown that the absence of anti-BP230 autoantibodies was an independent predictor of mucosal involvement in BP patients [53,59]. The presence of IgE anti-BP230 antibodies may also be clinically relevant. In a study by Hashimoto et al., IgE against BP180 was associated with disease severity, while anti-BP230 IgE was not. Conversely, in another study, the presence of anti-BP230 antibodies, but not of anti-BP180 antibodies, was found to be associated with the nodular prurigo-like phenotype of BP [59]. Because of the various methodological limitations of these reports, most invariably encompassing retrospective case series or single reports, these contentions need to be validated by adequate prospective studies encompassing larger numbers of BP patients with systematic comprehensive serological studies using different technical approaches.

## 7. Significance of BP230 to Neurological Disorders and BP

The epidemiological association between BP and several neurological diseases has been well confirmed in independent studies from different continents [60,61,62,63]. The exact reason responsible for this association between BP and conditions such as Parkinson’s disease, multiple sclerosis, and dementia remains unclear, although cross-reactivity between neuronal and epidermal antigens has been implicated. Using multivariate analysis, a large retrospective study of 273 BP patients showed that the presence of anti-BP230 antibodies, in addition to other variables, such as older age and female sex, was associated with the coexistence of neuropsychiatric disorders. After adjusting for confounders, anti-BP230 seropositivity proved to be the only independently significant predictor for comorbidity of neuropsychiatric diseases, such as dementia, epilepsy, multiple sclerosis, depression, and bipolar disorder in BP patients [64]. In another smaller study, BP patients with a comorbid neurological disease were found to have higher levels of both BP180 and BP230 autoantibodies, with a higher seropositivity rate for BP230 [65]. Although the BPAG1a/BPAG1b and BP230/BPAG1e isoforms share a homologous region, the existence of immunological cross-reactivity between these isoforms in patients with both BP and neurological disorders has not been yet convincingly demonstrated with well-performed experimental studies and adequate controls [36,66,67]. Furthermore, only a minority of the affected patients seem to possess autoantibodies directed against the shared plakin domain of BPAG1 [68]. The latter is usually not regarded as an immunodominant region in BP230/BPAG1e [8,10,12]. Finally, the expression pattern of BPAG1a in the central nervous system does not correlate with the various and heterogenous neurological diseases associated with BP [69].

## 8. BP230 Autoantibodies in Pruritus of the Elderly

Itch is almost invariably present and severe in patients with BP [70,71]. IgG autoantibodies against BP antigens and/or circulating autoantibodies directed against the epidermal basement membrane zone have been detected in a subgroup of elderly patients with various pruritic disorders by several approaches, such as immunoblotting, ELISA and/or indirect IF microscopy. These patients did not fulfill one of the key criteria for the diagnosis of BP, which is positive direct immunofluorescence studies. It is possible that some of these patients suffer from an early form of BP and later on develop the full-blown clinical picture of BP. In this context, it would be useful to carefully study, in a large prospective cohort, the dynamic of clinical features and of the immunological profile of patients presenting with pruritus and circulating autoantibodies against BP180 and/or BP230 but who do not show tissue-bound immunoreactants. Although the feasibility of such an observational and follow-up study is uncertain, the data gathered may provide new useful insights into the development of overt BP in paucisymptomatic patients at risk for BP [72,73,74,75,76,77,78,79,80]. Interestingly, IgG autoantibodies against BP230 seem to be more commonly detectable in patients with pruritic skin disorders than anti-BP180 antibodies [76,77,78,80]. In another report encompassing 112 BP patients, pruritus was associated with the presence of anti-BP230 IgG, but not of anti-BP180 IgG antibodies [81]. Feliciani et al. also found that 33% of elderly patients with pruritic disorders demonstrated antibody reactivity against either BP180 or BP230. Out of this group, 80% of patients showed reactivity to BP230 while 40% had antibodies against BP180 [77]. A recent study also identified a small subset of elderly patients with pruritus who had a similar autoantibody profile, with anti-BP230 being once again more frequently detected than anti-BP180 [74]. In this latter report, the authors also showed that some elderly patients with pruritus have autoreactive T-cells directed against the N- and C-domains of BP180. These autoreactive T-cells exhibited a cytokine profile similar to that typically observed in BP patients [82]. IgE antibodies against BP230, and to a lesser extent BP180, have also been found in pruritic elderly patients [83]. It is important to also note that age-related senescence of the skin, which includes impairments in physical and immunological barrier function, is thought to be a significant factor in the susceptibility of elderly patients to the development of BP in general [84].

When taken together, these findings underline our gaps in knowledge about the relationship between BP and the various chronic, pruritic, non-specific eczematous eruptions observed in the elderly. Itching and chronic scratching of the skin may cause cellular damage leading to the exposure and better recognition of intracellular and extracellular components, such as BP230 and BP180, which is then followed by loss of self-tolerance and triggering of a targeted humoral response. In this scenario, BP230 antibodies may develop following antigen unmasking in predisposed patients. Alternatively, circulating anti-BP230 antibodies may induce pruritic skin lesions which in turn may lead to overt BP. Following a prodromal phase, autoantibodies and an autoreactive Th2 response result in the development of overt BP [76]. In the early phase of the disease, with low levels of tissue-bound immunoreactants, the limited sensitivity of DIF may explain false-negative results.

## 9. Conclusions

BP230’s contribution to the pathogenesis of BP has gradually grown more nuanced. It is quite possible that anti-BP230 autoantibodies are sufficient to mediate the development of BP in affected patients. Recent experimental findings utilizing the adoptive transfer of splenocytes from BP230 immunized conditional BP230 knockouts provide significant support to a direct pathogenic mechanism [46]. The mechanism by which blistering is induced remains to be elucidated. For example, whether there is steric hindrance or alterations in the expression of key molecules in the hemidesmosome is unclear. While the development of autoantibodies to BP180 is thought to lead to intermolecular epitope spreading with subsequent development of anti-BP230 antibodies, do these anti-BP180 autoantibodies affect the pathogenicity of anti-BP230 antibodies? As the role of anti-BP180 antibodies targeting the NC16a domain in inducing macropinocytosis is well characterized, does this internalization regulate anti-BP230 endocytosis [85]? Additionally, the pathogenicity of anti-BP230 IgE autoantibodies is yet to be determined. Given its intracellular location, it is conceivable that it has a different mechanism of action on immune activation than anti-BP180 autoantibodies. Lastly, given the presence of anti-BP230 autoantibodies and the increased incidence of anti-BP180 antibodies, it remains to be determined whether these antibodies have a pathologic function, or are simply a result of antigen unmasking without a significant impact on itch.

## Data Availability

Not applicable.
